# Design of Heavy-Load Soft Robots Based on a Dual Biomimetic Structure

**DOI:** 10.3390/biomimetics9070398

**Published:** 2024-06-30

**Authors:** Liu Yang, Zhilei Zhang, Zengzhi Zhang, Yuzhong Lou, Shijie Han, Jiaqi Liu, Liu Fang, Shangsheng Zhang

**Affiliations:** 1 School of Mechanical and Electrical Engineering, China University of Mining and Technology (Beijing), Beijing 100083, China; 18811153693@163.com (L.Y.); cnefm_bj@163.com (Z.Z.); 15101667323@163.com (S.H.); 16636360420@163.com (J.L.); 2 School of Mechanical and Power Engineering, Dalian Ocean University, Dalian 116023, China; 18648508719@163.com (Z.Z.); l17866702216@163.com (Y.L.); 3 Department of Information Engineering, Ordos Institute of Applied Technology, Ordos 017000, China

**Keywords:** biomimetic machine, software machine, high load, flexibility, pneumatic drive

## Abstract

This study first draws inspiration from the dual biomimetic design of plant cell walls and honeycomb structures, drawing on their structural characteristics to design a flexible shell structure that can achieve significant deformation and withstand large loads. Based on the staggered bonding of this flexible shell structure, we propose a new design scheme for a large-load pneumatic soft arm and establish a mathematical model for its flexibility and load capacity. The extension and bending deformation of this new type of soft arm come from the geometric variability of flexible shell structures, which can be controlled through two switches, namely, deflation and inflation, to achieve extension or bending actions. The experimental results show that under a driving pressure within the range of 150 kpa, the maximum elongation of the soft arm reaches 23.17 cm, the maximum bending angle is 94.2 degrees, and the maximum load is 2.83 N. This type of soft arm designed based on dual bionic inspiration can have both a high load capacity and flexibility. The research results provide new ideas and methods for the development of high-load soft arms, which are expected to expand from laboratories to multiple fields.

## 1. Introduction

As a continuation of the development of bionic robots, soft robots still have the greatest advantage and design inspiration based on bionics [1,2]. At present, biomimetic soft robots have achieved various biomimetic movements such as crawling [3], jumping [4], swimming [5], wriggling [6], etc. [7,8], further enhancing the human understanding of the biological kinematics and biomechanics of soft tissues as well as structures. In addition, the flexibility of soft materials enables soft robots to have safe human–machine interactions [9] and a satisfactory environmental adaptability [10], which is expected to solve the shortcomings of traditional rigid robots that are difficult to apply in unstructured environments. In order to meet the application needs in different scenarios, researchers have developed various biomimetic soft robots, such as software end effectors for grasping irregularly shaped or fragile objects [11,12], soft medical robots for targeted drug delivery [13,14], and soft amphibious robots [15,16] used for monitoring coastal ecosystems. These soft robots have been successfully applied in fields such as biomimetic grasping and medical rehabilitation and have achieved good application results.

However, there are still challenges for soft robots safely manipulating objects in unstructured environments. Thanks to the inherent passive flexibility, continuous deformation, and interactive friendliness of soft materials, scholars have developed various new types of soft arms to address this long-term challenge. The current commonly used method is to use entanglement to restrict the radial expansion of cavity chambers or embed thin ropes and paper, etc., at specific positions in those cavity chambers to achieve local constraints [17,18] so as to change the internal cavity pressure difference in soft arms relying on fluid and control their movements [19]. In addition, there are soft arms that rely on pull wires [20] and shape memory alloys [21] to complete contraction actions, as well as pneumatic networks [22] and push rods [23] to complete extension actions. However, soft arms are limited by their insufficient load capacity when performing “manipulation” on objects.

In order to improve the comprehensive mechanical performance of soft arms, scholars have conducted in-depth research from the technical level of design torsion constraints [24], shell reinforcement [25], particle blocking mechanisms [26], 3D embedded printing [27], 4D printing [28], etc. In addition, some scholars, inspired by biomimetic inspiration in nature, have designed biomimetic structures that can withstand large loads, which are relied on to improve the loading capacity of soft arms. 

For example, inspired by the biomimetic design of the human shoulder structure, Lin [29] et al. developed lightweight and compact three-degrees-of-freedom membrane covering passive joints, which benefited from the static water stress of their filled liquid and achieved the open-loop control of three-degrees-of-freedom motions with a high load-bearing capacity in a compact space. Zhao [30] et al. proposed a soft, rigid hybrid intelligent artificial muscle based on a liquid crystal elastomer (LCE) and a spiral metal wire, inspired by the biomimetic design of human arms. Adopting the thermal responsiveness of LCEs and spiral metal wires, the requirements of a high load-bearing capacity and the electric heating function were met. Ruan [31] et al. proposed a ball joint with a continuously adjustable load-bearing capacity based on the principle of positive pressure friction. The proposed spherical joint can rotate freely in the atmosphere. Once the ball joint is driven to the reference position, the internal air chamber expands under a high pressure and locks the ball joint with a frictional torque to achieve a high load capacity. Liu [32] et al. directly coupled a rigid origami exoskeleton with a soft airbag and developed a new type of soft arm that could withstand a load of up to 5 kg during extension, contraction as well as bending movements.

However, due to the inherent properties of soft materials and manufacturing processes, soft arms with a high load capacity often have complex structures, difficult processes, as well as poor flexibility and stability. Unlike existing design methods, this work designs a modular flexible shell structure based on the dual biomimetic inspiration of plant cell walls and honeycomb structures. The soft arms prepared by interlocking and bonding such flexible shell structures have a high load capacity and flexibility. An advantage of this method is that the deformation of the prepared soft arms when filled with air pressure mainly comes from the geometric variability of the flexible shell structures, rather than relying on the linear expansion of the material itself, like most soft arms. Therefore, the soft arm proposed in this article can be prepared using materials with a higher Young’s modulus, without the need for complex processes to constrain specific positions or the use of rigid parts for an auxiliary design, and relies only on changes in the geometric shape of the flexible shell structure, ensuring flexibility while significantly improving the load capacity.

From the perspective of soft robots, this work provides a design method for soft arms combined with a high load capacity and flexibility, establishing a mathematical model for their flexibility and load capacity. From the perspective of engineering applications, this work has good modularity and applicability. Only by interlocking and bonding flexible shell structures can soft arms of different lengths and performances be manufactured, making up for the shortcomings of single types and poor mechanical performances. From the perspective of human–computer interaction scenarios, all of our soft arms are made of flexible materials, which can have stronger environmental adaptability and safety during task execution.

## 2. Materials and Methods

### 2.1. Flexible Shell Structure Design

As a continuation of the development of biomimetic robots, the design of soft robots and soft actuators mostly originated from soft organisms or organs in nature, such as octopuses, worms, elephant trunks, etc. Starting from bionics and simulating the movement mechanisms of soft organisms in nature, soft robots have been designed to achieve crawling, jumping, and other movements on land and under water.

**Figure 1:**
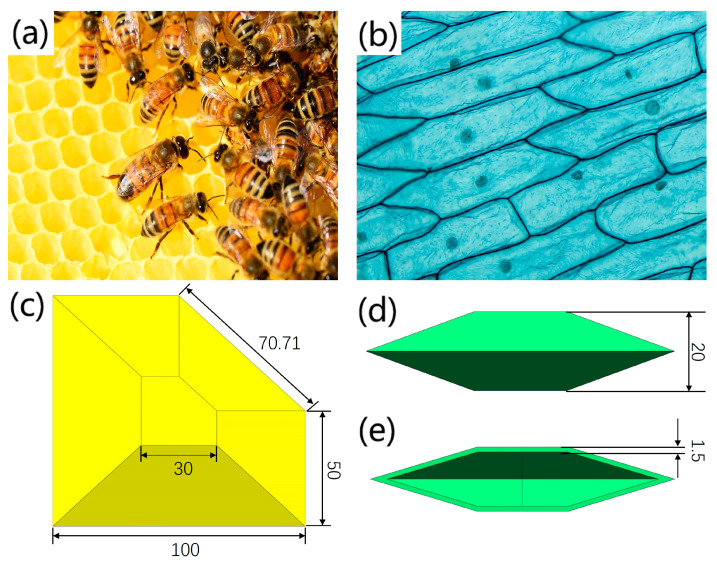
A flexible shell structure designed based on the dual biomimetic inspiration of plant cell walls and honeycomb structures (unit: mm). (**a**) Honeycomb structure; (**b**) Plant cell wall structure; (**c**) Overall structure of the flexible shell structure; (**d**) Side view of the flexible shell structure; (**e**) Section view of the flexible shell structure.

However, software machines designed with biomimetic inspiration from soft organisms have weak human output capabilities and may even be unable to carry their own gravity. However, in nature, polygonal honeycomb structures composed of several interconnected hexagonal units, as shown in Figure 1a, and the outermost cell wall structures of plant cells used to support and protect cells, as shown in Figure 1b, can improve structural strength while ensuring a light weight. The high strength of these structures is usually determined by the following:

1. A uniform material distribution: The material distribution of a honeycomb structure and a plant cell wall structure is relatively uniform, where each independent unit can withstand external loads. After the external load is effectively dispersed, the overall structure is subjected to more uniform forces.

2. Basic units are the same: The basic units of honeycomb structures and plant cell wall structures are polygonal shells. The orderly connection design of such unit structures can balance the forces among each unit and avoid areas with an excessive stress concentration.

Taking inspiration from the above biomimetic design, this article proposes a flexible shell structure that mimics the structure of a honeycomb and that of plant cell walls, which has a honeycomb structure shape on the front and a plant cell wall structure shape on the side, achieving a large deformation while withstanding large loads, as shown in Figure 1c. This spatial three-dimensional structure, composed of walls of an equal thickness and their internal cavities, has geometric variability. When its internal cavity is filled with air pressure, its geometric shape can be changed without causing any linear expansion of its own material, forcing deformation of the flexible shell structure. According to the actual scenario requirements, the deformation ability of the flexible shell structure can be controlled by changing the driving pressure of its internal cavity. It has two main functions: on the one hand, it provides a deformation space for the soft arm to extend and bend, and on the other hand, it preserves the flexibility and passive deformation of the material itself, protecting the environment and its own structure from damage in the event of accidents. Thanks to the above advantages, flexible shell structures can be prepared using materials with a relatively high hardness to improve their strength and stability, as well as expand their application range.

### 2.2. Soft Arm Design

**Figure 2:**
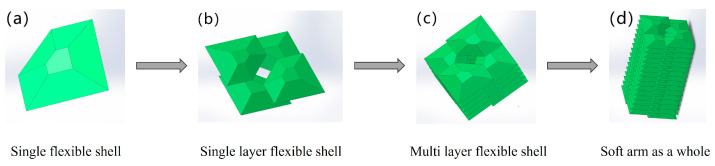
Preparation process of the soft arm. Apply a special soft adhesive to the trapezoidal plane and upper end face of the flexible shell structure and bond adjacent flexible shell structures. Repeat the above process until the soft arm is prepared.

This article proposes a design scheme for a large-load soft robot based on the interlocking bonding of a flexible shell structure. As shown in Figure 2, the soft arm consists of three segments. As shown in Figure 2d, the overall schematic diagram of the soft arm is prepared by interlocking and bonding fifteen layers of flexible shell structures. In Figure 2a, adjacent flexible shell structures are bonded at the trapezoidal plane of each flexible shell structure to form the first plane layer of the soft arm, as shown in Figure 2b. In the second plane layer, the interlocking bonding of five plane layers together forms one section of the soft arm, as shown in Figure 2c.

**Figure 3:**
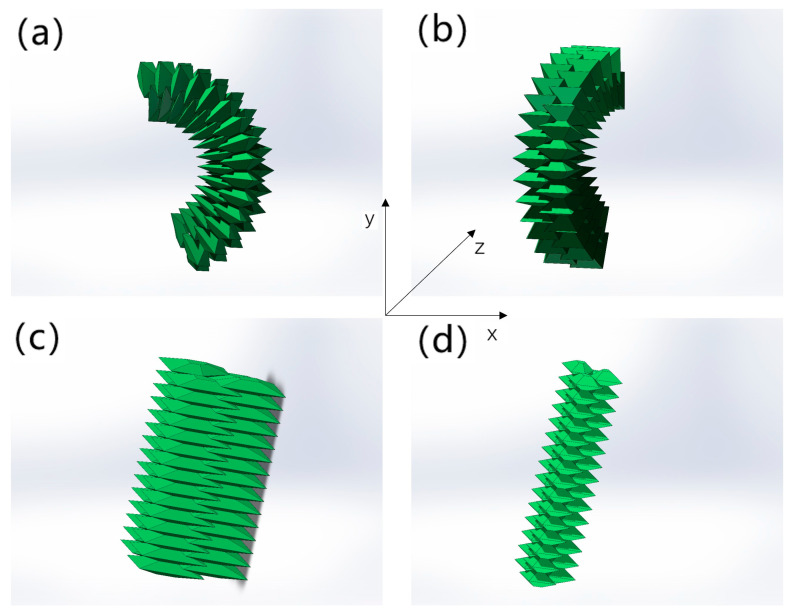
Schematic diagram of soft arm bending and elongation. (**a**) Different driving pressures input on the left and right sides of the soft arm; (**b**) The soft arm has different driving pressures for both front and back measurements; (**c**,**d**) The overall input of the soft arm is the same driving pressure.

When the flexible shell structure on one side of the soft arm is inflated, it will elongate under pressure, while the elastic deformation of that on the other side is relatively small, so the entire soft arm will bend towards the side with a lower pressure. Specifically, if different driving pressures are applied to the flexible shell structures on both sides, the soft arm will bend in the x-y plane, as shown in Figure 3a; if different driving pressures are applied to the flexible shell structures on both sides, the soft arm will bend in the y-z plane, as shown in Figure 3b. In addition, when both flexible shell structures are inflated simultaneously, the soft arm will extend along the z-axis, as shown in Figure 3c,d. By injecting different driving pressures into flexible shell structures at different positions, the soft arm can achieve different bending angles and elongation, achieving flexible deformations. The design of the flexible shell structure allows for radial deformations of the soft arm to be limited within a limited range. Therefore, soft arms can work at higher driving pressures to improve the load capacity.

### 2.3. Flexibility Model

The comprehensive performance of a soft arm mainly depends on its load capacity and the size of its executable task space. Therefore, it is crucial to establish a mathematical model for its load capacity and flexibility. In this article, we define flexibility as the amount of space that a soft arm can reach when its end is fixed at the other end, and the ratio of reachable spaces to the original length of the soft arm is defined as its relative flexibility. The size of the working areas of a soft arm in a plane refers to the areas that the tip of the arm can reach, and such spatial areas are axisymmetric, so only half of the areas can be calculated.

**Figure 4:**
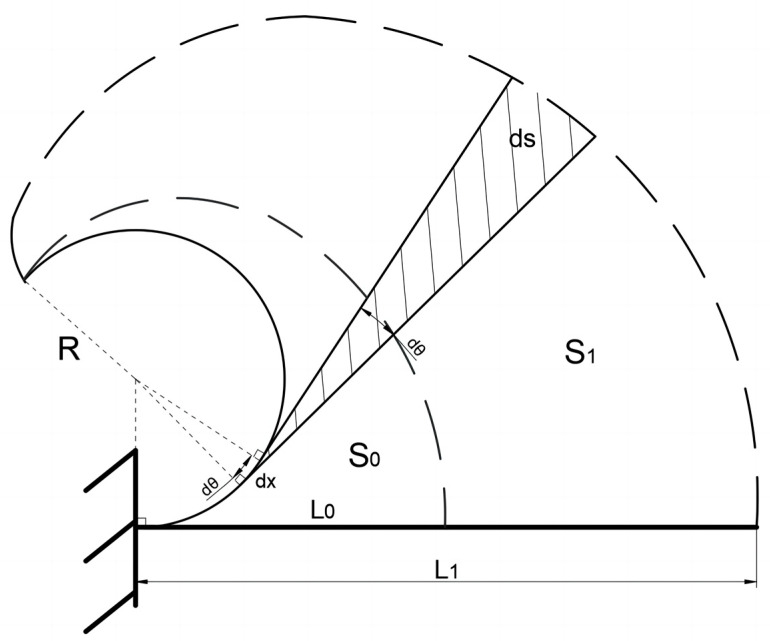
Flexibility calculation model for the soft arm.

Below is an approximate method for calculating the flexibility of the soft arm. The simplified model of the soft arm is shown in Figure 4. The outer dashed line in the figure represents the path through which the tip of the soft arm passes during the bending process from the maximum elongation state (driving pressure of 100 kpa) to the ultimate state (the upper flexible shell structure pressure is zero). The specific implementation process of the physical object is to first fill all the flexible shell structures in the soft arm with the maximum air pressure and then slowly deflate the flexible shell structure on the upper side of the soft arm. The inner dashed line in the figure represents the path through which the tip of the soft arm passes during the bending process from the initial state (with zero driving pressure) to the ultimate state (with a pressure of 100 kpa inside the lower flexible shell structure). The specific implementation process of the physical object is to slowly inflate the flexible shell structure on the lower side of the soft arm until the maximum air pressure is reached. We approximately assume that the area enclosed by these two dashed lines and the axis of the arm is half of the working area of the soft arm.

To calculate the area of this region, we first calculate the area enclosed by the inner and outer dashed lines as well as the circular arc with the ultimate bending radius R in the figure, denoted as $S_{0}$ and $S_{1}$, which are then subtracted to obtain half of the working area of the soft arm. We represent the maximum bending radius, the original length, the maximum length and the width of the soft arm as R, $L_{0}$, $L_{1}$, and D Respectively. For more accurate calculation results, we choose the trajectory of two lines representing the inner and outer boundaries, where the length of the soft arm remains approximately unchanged during the bending process. These two lines are located in the staggered area of the upper and lower flexible shell structures, approximately at a distance of $\frac{D}{3}$ from the centerline. Therefore, the bending radius of the inner and outer boundary lines is:

$$R_{0}=R-\frac{D}{3},R_{1}=R+\frac{D}{3}$$

In order to calculate the area of the two regions separately, we use the differentiation method to divide the area into small fan-shaped units of dS:

$$dS_{i}=\frac{1}{2}{\left(L_{i}-X_{i}\right)}^{2}d\theta_{i},d\theta_{i}=\frac{dX_{i}}{R_{i}},(i=0,1)$$

Then, through integration operations, it can be obtained as

$$S_{i}=\int_{0}^{\frac{L_{i}}{R_{i}}}\frac{1}{2}{\left(L_{i}-X_{i}\right)}^{2}d\theta_{i}=\frac{{L_{i}}^{3}}{6R_{i}},(i=0,1)$$

Subtracting $S_{0}$ and $S_{1}$ yields half of the working area of the soft arm. Then, the entire work area is divided by the original length of the soft arm to obtain the relative flexibility, represented by f:

$$f=\frac{2\left(S_{1}-S_{0}\right)}{L_{0}}=\frac{1}{3L_{0}}\left(\frac{{L_{1}}^{3}}{R+\frac{D}{3}}-\frac{{L_{0}}^{3}}{R-\frac{D}{3}}\right)$$

### 2.4. Load Capacity Model

This article defines load capacity as the maximum load moment when a soft arm can maintain stability with its load-bearing end at the same height as the fixed end. The load capacity of a soft arm is defined in this article as the maximum load moment when it can maintain stability with its load-bearing end at the same height as the fixed end. There are countless ways for a soft arm to reach the same horizontal position as the tip and base under load. In order to simplify the problem, this article chooses to only input a driving pressure to the lower flexible shell structure of the soft arm when modeling and analyzing its load capacity.

**Figure 5:**
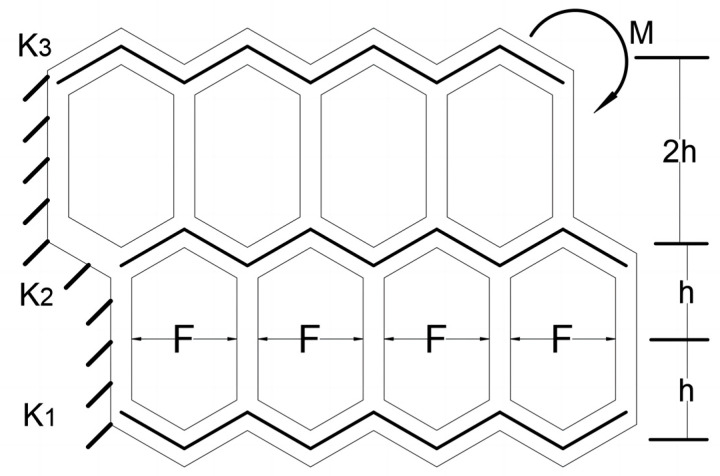
Analysis model of the load capacity of the soft arm.

In this case, we introduced a simplified model to analyze the load capacity of the soft arm. The tangential bearing capacity of this model is better, and its tangential strain is smaller due to the ideal mechanical properties of the biomimetic structure of the flexible shell structure. Therefore, we ignore the tangential deformation of the soft arm and only consider the stress and deformation in its extension direction. As shown in Figure 5, we assume a polygon with a flexible shell structure height of 2h, and the staggered parts (with internal lines) are simplified as springs, $k_{1},k_{2},\;and\;k_{3}$ represent the spring coefficients. F represents the internal force provided by the cavity inside the flexible shell structure, determined by the contact area S and the pressure P. M represents the load torque provided at the tip of the soft arm.

We assume that the elongation of the flexible shell structure at the equilibrium is Δs. According to the force system equilibrium, the following equation can be listed:

$$F=2(k_{1}+k_{2}+k_{3})\Delta s$$

The load torque can be calculated according to the following formula:

$$M=Fh-4hk_{1}\Delta s+4hk_{3}\Delta s=Sph\left(1+\frac{2\left(k_{3}-k_{1}\right)}{k_{1}+k_{2}+k_{3}}\right)$$

From the above theoretical model, we can conclude the following:

If the soft arm is a symmetrical structure (i.e., $k_{1}=k_{3}$), its maximum load capacity is Sph. In addition, its load capacity can be improved by increasing the air pressure, flexible shell structure height, and cavity area; if the soft arm is an asymmetric structure ($k_{3}>k_{1}$), its load capacity may be greater than Sph, which can be improved by increasing ($k_{3}-k_{1}$). In theory, when $k_{3}\to \infty$, the load capacity of the soft arm can reach its maximum limit load of 3Sph.

## 3. Results

### 3.1. Prototype Preparation

**Figure 6:**
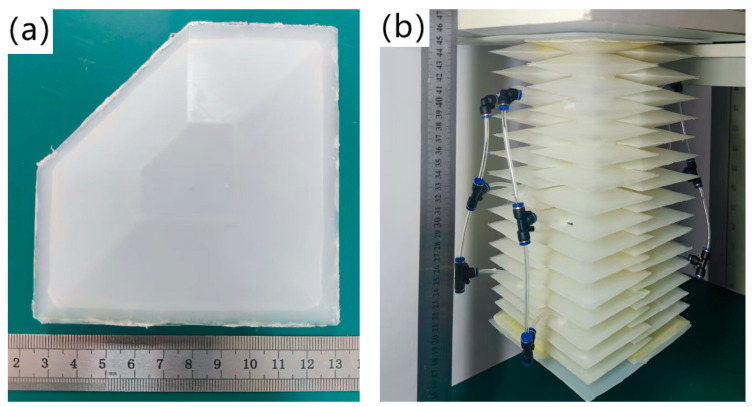
Physical image of the prototype. (**a**) Physical drawing of flexible shell structures; (**b**) Physical image of the soft arm.

The body material of the flexible shell structure is selected as HC9020 silicone, and its preparation is completed using the mold casting method, as shown in Figure 6a. Finally, a silicone special adhesive is used to prepare the soft arm through hand processing and interlocking bonding. Silicone hoses and pneumatic tees are used to connect each column of the flexible shell structures of the soft arm together as a set of driving units, as shown in Figure 6b. Among them, a pouring mold is modeled and designed using SolidWorks before 3D printing. R4600 resin is selected as the printing material, which has a good printing accuracy and stability. The surface of the mold is sprayed with the Smooth on Release 200 aerosol release agent. After pouring the silicone material into the mold, it is placed in an oven for constant-temperature curing for 2 h before being removed.

**Figure 7:**
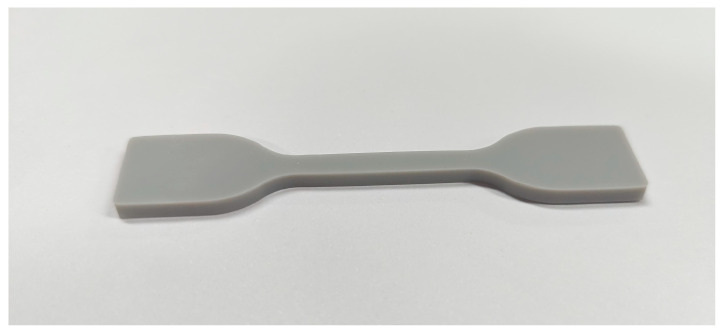
Dumbbell-shaped specimen.

According to the relevant provisions of the national standard GB/T528-2009, namely, *Determination of Tensile Stress-strain Properties of Vulcanized Rubber or Thermoplastic Rubber*, we prepared the material of the soft robot body into a type II dumbbell-shaped specimen for uniaxial tensile testing, as shown in Figure 7.

The uniaxial tensile test was conducted using an HZ-1004B testing machine produced by LiXian Instrument Technology Co., Ltd. The upward tensile test was conducted at a speed of 500 mm/min until the sample broke, with the real-time tensile strength and corresponding silicone style strain recorded. The maximum force value, tensile strength, and elongation when the test sample broke are shown in Table 1.

The tensile strength of the flexible shell structure material is 7.822 Mpa by the tensile test. After the finite element analysis and physical verification of the flexible shell structure, the maximum aeration pressure is determined to be 214 kpa.

### 3.2. Flexibility Experiment

**Figure 8:**
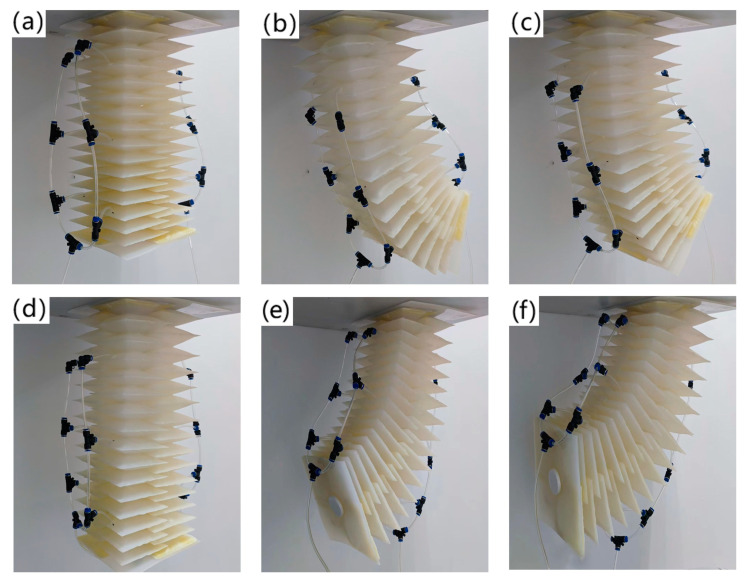
The flexibility experiment process of a soft robot. (**a**) No input of driving pressure; (**b**) Left driving pressure 150 kpa, right driving pressure 50 kpa; (**c**) Left driving pressure 150 kpa, right driving pressure 0 kpa; (**d**) Left driving pressure 150 kpa, right driving pressure 150 kpa; (**e**) Left driving pressure 50 kpa, right driving pressure 150 kpa; (**f**) Left driving pressure 0 kpa, right driving pressure 150 kpa.

The flexibility experiment process of the soft arm is as follows: fix the base of the soft arm and record its original length. Starting from the fixed end of the arm to its tip, slowly input a driving air pressure to its left flexible shell structure until the pressure reaches 150 kpa, and then stop inflating. At this time, the soft arm moves to the right boundary of its executable task area, and the movement trajectory of its tip is the inner boundary of its executable task area; the driving air pressure is slowly input into the flexible shell structure on the right side of the soft arm until the pressure reaches 150 kpa, and then inflation is stopped. At this time, the soft arm moves to the farthest position of its executable task area, and the movement trajectory of its tip is the outer boundary of its executable task area; then, the flexible shell structure on the left side of the soft arm is slowly deflated until the internal pressure reaches zero. At this point, the soft arm moves to the left boundary of its executable task area, and the movement trajectory of its tip is the outer boundary of its executable task area; finally, the flexible shell structure on the right side of the soft arm is slowly deflated until the pressure reaches zero, and the movement trajectory of its tip is the inner boundary of its executable task area. As shown in Figure 8, under a driving pressure of 150 kpa, the maximum elongation of the soft arm reaches 23.17 cm, and the maximum bending angle is 94.2 degrees.

When conducting the flexibility experiment on the soft arm, a red marker is attached to the tip of the arm, and the tip of the marker is in contact with the white background paper. After performing a complete motion cycle, the motion path of the arm tip was recorded by a red marker on a white background paper, and this area was calculated using the Monte Carlo method. The L_1_, R, and arm design parameters L_0_ and D obtained in the soft arm experiment are introduced into the flexibility theoretical model, and the results are calculated. It is found that the theoretical calculation area is only 1.8% larger than that obtained by the Monte Carlo method. Therefore, we believe that the theoretical model of flexibility is correct.

### 3.3. Load Capacity Experiment

**Figure 9:**
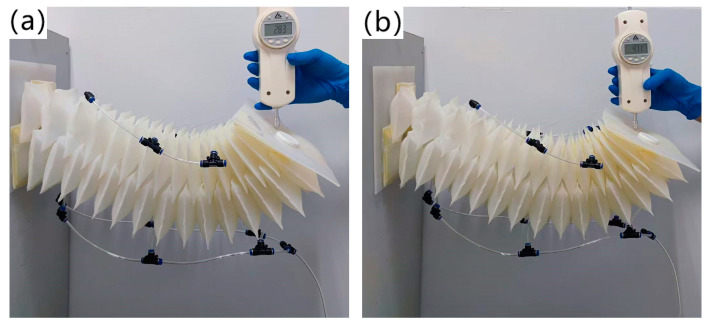
Load capacity experiment of soft robots. (**a**) The effective load force of the soft arm under a driving pressure of 150 kpa is 2.83 N; (**b**) The effective load force of the soft arm under a driving pressure of 200 kpa is 4.71 N.

The experimental process of the load capacity of the soft arm is as follows: input a driving air pressure to the flexible shell structure on the lower side of the soft arm, apply a slowly increasing load force at its end until the fixed end and the end of the soft arm reach the same horizontal height, and record the load force of the soft arm and use it as the maximum load capacity. As shown in Figure 9a, the maximum load force of the soft arm under a driving pressure of 150 Kpa is 2.83 N, but the load capacity obtained from this experimental scheme is a conservative value. In practical applications, inputting higher driving pressures to flexible shell structures can effectively improve the load capacity of soft arms. As shown in Figure 9b, the maximum load capacity of the soft arm increases to 4.71 under a driving pressure of 200 kpa, which is much greater than its maximum load capacity measured under a driving pressure of 150 kpa.

## 4. Conclusions

In this study, our flexible shell structure design comes from the dual bionic inspiration of plant cell walls and honeycomb structures, and the soft arm prepared based on the staggered bonding of such flexible shell structures has a high load capacity and flexibility. Compared with flexible arms relying on the linear expansion of their own material to achieve flexible deformations, the bending and elongation deformations of our new soft arm rely on the geometric variability of the flexible shell structure; meanwhile, the manufacture of the flexible shell structure and the assembly process of the soft arm are simple. Compared with soft arms relying on other interference technologies to increase the load capacity, our software arm has a stronger environmental adaptability and safety in the application of human–computer interaction scenarios.

It should be pointed out that the design method proposed in this article, which combines flexibility with load capacity, as a universal method, only requires the staggered bonding of flexible shell structures to manufacture soft arms with different lengths and performances, making up for the shortcomings of single types and poor mechanical performances, which gives it the potential to expand to a wider range of soft robot fields. In practical use, soft arms prepared based on the staggered bonding of flexible shell structures can also reduce the cost of failure (after the soft arms expand and explode, only the corresponding positions of the flexible shell structures need to be replaced). For general tasks, the extension or bending of soft arms can be achieved through two switches: deflation and inflation.

In this article, the soft arm is now only prepared by bonding fifteen layers of flexible shell structures, with limited degrees of freedom of movement. In future research, more degrees of freedom can be increased by extending the length of soft arms. Meanwhile, extended soft arms will shorten the force arms when bent in the middle, allowing them to withstand greater loads.

Due to the convenience of the preparation process, the proposed soft arm structure can have different expected sizes. Future research will establish the relationship between the comprehensive performance of soft arms and design parameters (wall thickness and shape), providing theoretical guidance for the preparation of the prototypes. According to different application scenarios, a soft arm that can meet usage requirements without causing excessive performance can be prepared.

The current prototype is limited by not being equipped with satisfactory end effectors, which only demonstrates the application potential of soft arms through simple command programming. The command programming at this time is achieved by manually setting the inflation and deflation time as well as the inflation pressure of each group of airbags, without control. In future work, we will design end effectors with different structures to be used in conjunction with soft arms for different tasks, and the control system of soft arms will also be developed using learning methods similar to neural networks.

## Figures and Tables

**Table 1:** Material parameters of soft robots.

Index	Parameter
Density g/cm^3^	1.3
Maximum tensile force N	80.257
Tensile strength Mpa	7.822
Breaking elongation rate%	280.409

## Data Availability

All data are available in the main text.
